# Role of MicroRNAs in Renin-Angiotensin-Aldosterone System-Mediated Cardiovascular Inflammation and Remodeling

**DOI:** 10.1155/2015/101527

**Published:** 2015-05-06

**Authors:** Maricica Pacurari, Paul B. Tchounwou

**Affiliations:** ^1^Biology Department, College of Science, Engineering and Technology, Jackson State University, Jackson, MS 39217, USA; ^2^NIH RCMI-Center for Environmental Health, College of Science, Engineering and Technology, Jackson State University, Jackson, MS 39217, USA

## Abstract

MicroRNAs are endogenous regulators of gene expression either by inhibiting translation or protein degradation. Recent studies indicate that microRNAs play a role in cardiovascular disease and renin-angiotensin-aldosterone system- (RAAS-) mediated cardiovascular inflammation, either as mediators or being targeted by RAAS pharmacological inhibitors. The exact role(s) of microRNAs in RAAS-mediated cardiovascular inflammation and remodeling is/are still in early stage of investigation. However, few microRNAs have been shown to play a role in RAAS signaling, particularly miR-155, miR-146a/b, miR-132/122, and miR-483-3p. Identification of specific microRNAs and their targets and elucidating microRNA-regulated mechanisms associated RAS-mediated cardiovascular inflammation and remodeling might lead to the development of novel pharmacological strategies to target RAAS-mediated vascular pathologies. This paper reviews microRNAs role in inflammatory factors mediating cardiovascular inflammation and RAAS genes and the effect of RAAS pharmacological inhibition on microRNAs and the resolution of RAAS-mediated cardiovascular inflammation and remodeling. Also, this paper discusses the advances on microRNAs-based therapeutic approaches that may be important in targeting RAAS signaling.

## 1. Introduction

The role of microRNAs in RAAS system is at early stages of investigations; however, few microRNAs have been shown to be implicated in the RAAS mediated hypertension cardiovascular diseases [1]. Blocking RAAS is a primary approach for the treatment of hypertension, cardiovascular inflammation, and cardiac hypertrophy [2]. The discovery of microRNAs in 1993 in nematode* Caenorhabditis elegans* has led to a new research avenue and provided novel and innovative tools to understand gene regulation that sometimes could not be explained. Since then, more than 2,518 microRNAs have been identified and listed in current databases [3]. Angiotensin II (Ang II) is the main active effector of the RAAS with profound signaling effects on the cardiac and vascular systems. Ang II impacts the cardiovascular system particularly regulating the proliferation and migration of vascular smooth muscle cells (VSMC) therefore affecting cardiovascular remodeling. Ang II signaling is mediated via Ang II type I receptor (ATIR), and both the Ang II and ATRI are highly expressed in the VSMC of some of cardiovascular disease (CVD). In addition to Ang II, tumor necrosis factor alpha (TNFalpha) plays an important role in the development of cardiovascular inflammation, sometimes in tandem with Ang II. MicroRNAs regulate many important biological functions and abnormal levels of microRNAs are involved in cardiovascular and other pathologies.

In this review, we attempt to provide information of microRNAs that have been shown to play a role in the RAAS signaling and cardiovascular inflammation/remodeling and related CVD.

## 2. MicroRNA Biogenesis and Stability

The main function of microRNA is to bind to 3′ UTR of its target gene and suppress its expression. MicroRNAs are conserved small noncoding double-stranded strands of RNA of approximately 22 nucleotides in length. Gene regulation via microRNAs presents some level of complexity given that microRNA can be part of a coding and noncoding gene and can be independently expressed or can form a cluster sharing same transcriptional regulation [4]. Furthermore, the complexity of microRNAs signaling is extended by the finding that microRNAs are multifunctional as such one microRNA can bind to multiple targets, and more than one microRNA can bind to the same 3′ UTR [5].

MicroRNAs biogenesis is a complex and important step in microRNA activity. Biogenesis of microRNAs is under temporal and spatial control, involving an intricate coordination of proteins, transcription factors, cofactors, and RNA [6]. In addition to microRNAs regulation by Drosha and Dicer proteins, additional levels of modification processes such as editing, methylation, uridylation, adenylation, or even RNA decay are emerging as key factors in regulation of microRNA biogenesis [7]. MicroRNAs abundance is dependent on the presence of Argonaute proteins. It has been previously reported that a loss of Ago2 resulted in loss of microRNA and the reexpression of Argonaute proteins led to increased expression of precursor microRNAs [8]. However, the mechanisms that regulate microRNAs turnover are not fully understood neither perhaps fully identified. Of all aspects of microRNAs, stability is one major property that makes microRNAs powerful tools in cell biology. MicroRNAs are stable in many biological fluids including circulating blood, urine, and breast milk [9]. Moreover, microRNAs can be found encapsulated in vesicles but also there are microRNAs that are not nonencapsulated but bound to other circulating macromolecules and account for majority (~80%) of circulating microRNAs [10]. Due to their stability, many microRNAs are considered potential biomarkers of several diseases, including cardiovascular diseases.

## 3. MicroRNA and RAAS Effectors

Recent estimates suggest that one-third of all genes are regulated by microRNAs. In mouse primary cultured VSMC, overexpression of miR-155 inhibited Ang II-induced cell proliferation and viability via decreasing ATIR mRNA and protein [11]. Numerous studies showed that miR-155 plays an important role mediating inflammatory and immune responses and hematopoiesis [12]. However, miR-155 is also highly expressed in numerous types of cancer, and thus it seems that miR-155 may indeed regulate diverse biological functions [12]. Alexy and coworkers examined the formation of miR-155 encapsulated microvesicles (MP) by endothelial cells (EC) following TNFalpha treatment. In the presence of TNFalpha, EC released a higher level of miR-155/MP but tremendously decreased the level of miR-126 and miR-21/MP. The TNFalpha-induced miR-MP exerted antiapoptotic effect, whereas the low miR-MPs were proapoptotic. These results suggested also a role of microRNAs in cell to cell communication signaling pathway [13]. MiR-155 plays a key role in mediating cardiac injury, cardiac remodeling, and inflammation in hypertensive or pressure overload heart via regulating AT1R, eNOS, and inflammatory cytokines. In aortic adventitial fibroblast, miR-155 regulates AT1R [14]. Overexpression of miR-155 decreased the expression of AT1R and prevented Ang II-induced ERK1/2 activation and increased the expression of *α*-smooth muscle actin (*α*-SMA) [14]. Moreover, miR-155 targets endothelial nitric oxide synthase (eNOS), thus directly regulating endothelium-dependent vasorelation [15, 16]. Patients with nephrolithiasis exhibited high levels of miR-155 in blood and urine [17]. Urine MiR-155 level negatively correlated with IL-6, IL-1*β*, IL-6, and TNF-*α* and positively with RANTES [17]. Another level of intricacy between RAAS, microRNA, exercise, and hypertension was explored by Sun et al. [15]. In this study, exercise attenuated aortic remodeling and improved endothelium-mediated vasorelaxation in SHR rats. Exercise increased miR-27a and miR-155 and decreased miR-143. Exercise also reduced Ang II level, increased Ang (1–7) levels, ACE2, AT2R, and Mas receptors, and suppressed ACE a target of miR-27a and AT1R which is a target of miR-155. This study provided an insight into the possible mechanism by which exercise improves RAAS in aorta and might explain the beneficial effect of exercise on cardiovascular system [15].

Ang II plays an important role in vascular remodeling by increasing the expression of TGFbeta, Col1A1, and alpha-smooth muscle actin (*α*-SMA). Pan et al. examined the effect of Ang II on miR-29b expression in the kidney of spontaneously hypertensive rats (SHRs). Ang II decreased the expression of miR-29b in the renal cortex of SHRs and in NRK-52E treated cells. In NRK-52E cells, miR-29b targets TGFbeta and *α*-SMA, and Col1A1, Col3A1, and overexpression of miR-29b abolished Ang II-induced genes [18]. In HEK293N cells overexpressing AT1R, Ang II increased miR-132 and miR-212 via AT1R/G*α*q/ERK1/2-dependent axis. In primary cardiac fibroblast, Ang II induced the expression of miR-132 and miR-212 in the heart, arteries wall, and kidney but no Ang II effect on these microRNAs in primary myocytes [19]. In hypertensive rats, Ang II induced the expression of miR-132 or miR-212. Moreover, patients taking AT1R blockers (losartan, candesartan, irbesartan, and telmisartan) exhibited decreased levels of miR-132 and miR-122 [20]. Both miR-132 and miR-212 are highly conserved miRNAs, closely clustered and regulated by cAMP response element binding protein (CREB), which is Ang II target gene. In most tissues, the level of miR-132 is much higher than that of miR-212, and the exact role of such difference is not known; however, it is proposed that miR-132 may indeed have a regulatory effect on miR-212 [21]. Overexpression of miR-132/212 in fibroblasts resulted in differential expression of 24 genes of which 7 genes (AGTR1, AC, PKC, EGR1, JAK2, cJUN, and SOD2) are involved in Ang II signaling. Functionally, overexpression of miR-132/212 induces increased fibroblast size and increased expression level of Ang II. Among the modulated genes, DYRK2 and MAP3K3 were found to be downregulated and known to be involved in endothelial to mesenchymal transition [22]. These results suggested that miR-132/212 regulates many genes of Ang II signaling pathway [19] (Table 1).

In patients with renal carcinoma, miR-129-3p and miR-129-5p were significantly attenuated compared to normal biopsy specimens. Moreover, ectopic expression of miR-129-3p inhibited cell migration and invasiveness, whereas renal carcinoma cells treated with miR-129-3p resulted in decreased level of metastasis genes including SOX4, phosphorylated focal adhesion kinase (FAK), and MMP-2/MMP-9 [23].

Recent studies have shown Ang II role in epithelial-mesenchymal transition (EMT), and microRNA role in such process was observed by Pan et al. [18] in spontaneously hypertensive rats (SHRs) and age-matched Wistar-Kyoto (WKY) rats. MiR-29b in renal cortex was lower in SHR than in WKY rats, and treatment of NRK-52E renal tubular epithelial cells with Ang II decreased miR-29b and increased expression of TGFbeta, *α*-smooth muscle actin (*α*-SMA), and collagen I (Col I). Mir-29b is emerging as microRNA associated with EMT [24] (Table 1). Li et al. [24] showed that TGFbeta downregulated miR-29b, whereas overexpression of miR-29b blunted TGFbeta-induced EMT via AKT2. Inhibition of miR-29b resulted in the expression of EMT markers.

Aldosterone synthase (Cyp11B2 gene) is a target of Ang II and thus a target of Ang II regulated microRNAs. Cyp11B2 gene is a target of miR-766 in human adrenocortical cells H295R [25]. Maharjan et al. [25] showed that miR-766 binds to Cyp11B2 gene and reduces Cyp11B2 mRNA and protein level. The findings of this study are intriguing since microRNAs regulate protein expression; however, this study suggests that microRNA also affects mRNA of its target.

## 4. MicroRNA and RAAS Inhibitors

The effect of RAAS inhibition on microRNAs was investigated by Deiuliis et al. in patients with atherosclerosis plaque progression [26]. Patients were given aliskiren for 12 weeks and peripheral blood mononuclear cells were collected and microRNAs arrays were performed. Aliskiren-treated patients had significantly downregulated miR-106b-5p, miR-27a-3p, and miR-18b-5p compared to placebo-treated patients. The level of microRNAs positively correlated with thoracic and abdominal aorta wall in patients treated with Aliskiren. In a different clinical setting such as in patients with acute stroke, plasma miR-106b-5p was found to be highly elevated compared to healthy patients [27]. Although the function of miR-106b-5p is not known yet, these findings suggest that miR-106-5p may play a role in hemodynamics. MiR-27a-3p has been shown to regulate EGFR/AKT1/mTOR axis thus to decrease cell viability and increase apoptosis, whereas overexpression of EGFR, AKT, or mTOR decreases miR-27a-3p-induced cell viability [28]. To identify angiotensin II (Ang II) regulated microRNAs, Kemp et al. performed genome-wide microarrays analysis in vascular smooth muscle cells treated with Ang II or losartan [29]. A high number of microRNAs (468) were regulated by Ang-II and losartan. Only 32 microRNAs were regulated by Ang II/AT2R, whereas 52 miRNAs were regulated via AT1R and 18 microRNAs were commonly regulated via AT1R and AT2R. Of all microRNAs, miR483-3p expression was significantly downregulated in response to chronic activation of AT1R. AT1R antagonist candesartan significantly increased miR-483-3p. Kemp et al. [29] also shed some insight on Ang II feed-forward regulation of RAAS effectors AGT, ACE-1, ACE-2, and AT2R via miR483-3p. In the presence of Ang II, miR483-3p is depressed, whereas RAAS effectors are highly expressed via 3′UTR binding sites of miR483-3p present on RAAS effectors [29]. A recent study of patients with coronary artery disease (CAD) receiving ARB, ACEI, and statins for 12 months provided evidence of Toll-like receptor 4 (TLR-4) regulated microRNAs. Four microRNAs including miR-31, miR-181a, miR-16, and miR-145 were downregulated in CAD patients compared to non-CAD patients. The treatment combination of ARB telmisartan and atorvastatin or ACEI enalapril and atorvastatin increased the TLR-4 responsive microRNAs and decreased TLR-4 protein level. ARB treatment induced a greater change of the four microRNAs compared to ACEI [30]. Another microRNA, miR-146a/b, was found at high levels in the blood of CAD patients, and its expression positively correlated with IRAK, TRAF, TLR4 mRNA, or protein [31]. After 12 months of treatment with atorvastatin and telmisartan or atorvastatin and enalapril, miR-146a/b, IRAK, TLR4 mRNA, or protein decreased in the blood of CAD patients. Correlation analysis revealed that miR-146-a and TLR4 were independent predicators of cardiac events [31] (Table 2).

## 5. MicroRNA in Cardiovascular Disease

Cardiovascular disease (CVD) still remains the major cause of worldwide death, and identifying new molecular factors with roles in the development of CVD may offer novel diagnostic markers for cardiovascular events. In patients with atypical coronary artery disease, a signature of five microRNAs miR-487a, miR-502, miR-208, miR-215, and miR-29b was found to be altered and thus may be considered potential novel diagnostic biomarkers [32]. Molecular targets for several of the five microRNAs were found to be mediators of local inflammation, such as miR-215 targets catenin-beta interacting protein 1 in TGFbeta stimulated rat mesangial cells, whereas miR-29b plays an important role in modulating myocardial injury and idiopathic fibrosis [33–35]. MiR-29 family regulates extracellular matrix proteins and thus also influences remodeling. Potential therapeutic applicability of miR-29 has been experimentally tested in the settings of induced pulmonary fibrosis. In the bleomycin-induced pulmonary fibrosis, treatment with miR-29 reversed fibrosis by decreasing collagen (Col1A1 and Col3A1) synthesis. Moreover, tissue analysis revealed the presence of intravenously injected miR-29b not only in the lungs but also in the cardiac muscle and spleen [35].

**Figure 1:**
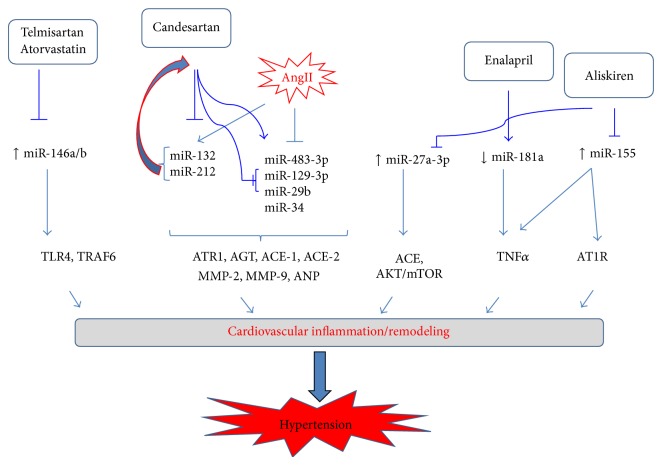
Dependent and independent RAAS-regulated microRNAs signaling in cardiovascular inflammation/remodeling and hypertension. Ang II regulates its level via stimulating miR-132, miR-212, and its downstream signaling via suppressing miR-483-3p, miR-129-3p, miR-29b, and miR-34 by increasing the expression of AT1R, AT2R, ACE1, ACE2, Col1A, and TGFbeta. Several microRNAs regulate RAAS signaling independent of Ang II via regulating inflammation and remodeling miR-146a/b, miR-181a, miR-155, miR-129-3p, and miR-29b. RAAS inhibitors differentially regulate microRNAs: telmisartan, atorvastatin, aliskiren, and candesartan inhibit miR-146a/b, miR-132, miR-212, miR-155, miR-129-3b, and miR-29b. Enalapril stimulates the expression of miR-181a which targets TNF*α* therefore regulating inflammation and remodeling. ↑: increased level, ↓: decreased level; ⊥: inhibition, and →: stimulation. AT1R: angiotensin II type 1 R; ACE: angiotensin converting enzymes; AGT: angiotensinogen; TLR4: toll-like receptor 4; TRAF6: TNF receptor associated factor 6.

In a different cardiovascular pathology such as in patients with failing heart, ischemic cardiomyopathy, or aortic stenosis, miR-320 was found to be highly expressed compared to control patients [36]. The functional analysis of miR-320 via ectopic expression in cultured cardiomyocytes indicated that miR-320 regulates cell death and apoptosis gene [37]. MicroRNA analysis in the blood and cerebrospinal fluid (CSF) of patients that suffered a stroke showed a differential profile of the two tissues, and hence some microRNAs were absent in one tissue but present in the other. In the CSF 183, microRNAs were detected out of which let-7c and miR-221-3p were upregulated and correlated with stroke. Analysis of blood showed a higher number of detected miRNAs a total of 287 out of which miR-151a-3p and miR-140-5p were upregulated and miR-18b-5p was downregulated and correlated with stroke [26]. Also, patients with atherosclerosis and receiving aliskiren for 12 weeks had a decreased blood level of miR-18b-5p, miR-106b-5, and miR-27a-3p [38]. Although both cardiovascular diseases, stroke and atherosclerosis, are due to blood clots formation, some microRNAs might just be disease specific, as, for example, miR-18b-5p is decreased in the blood of stroke patients but not in patients with atherosclerosis [26, 38] (Table 2). Recent studies support microRNAs role in cardiac hypertrophy [22]. For example, inhibition of miR-1, miR-23a, and miR-133 increased cardiomyocytes hypertrophy, whereas miR-22 or miR-30a regulates cardiac hypertrophy in mice [39–43]. MicroRNA signaling is complex; for example, one microRNA can target multiple genes. MiR-34 targets cell cycle genes and cardiac autophagy [44]. In addition to microRNAs modulating cardiomyocytes, Ang II is also a regulator of cardiomyocytes hypertrophy [45]. With regard to this relationship, Huang et al. [45] have shown that Ang II-induced myocardial hypertrophy was antagonized by miR-34, whereas inhibition of miR-34 promoted Ang II signaling via ANP and *β*-MHC [46]. Another microRNA regulating cardiomyocytes hypertrophy is miR-16 [16, 47]. Huang et al. [47] showed that overexpressing miR-16 in cardiomyocytes decreases Ang II, whereas overexpressing miR-16 resulted in decreased expression of cyclins D2, D2, and E in the myocardium of mice. As shown in Figure 1, based on the existing experimental evidence, microRNAs and RAAS signaling are complex particularly such that RAAS effector Ang II coregulates its level via microRNA-132 and microRNA-212 which also targets Ang II signaling via AT1R. RAAS inhibitors mostly target microRNAs by suppressing their expression thus alleviating cardiovascular inflammation and remodeling.

## 6. Conclusion

Considering the fact that millions of people worldwide are affected by hypertension and knowing the role played by RAAS in cardiovascular inflammation and remodeling, the determination of microRNAs role in regulating RAAS signaling may represent a new strategy in the development of novel therapeutics as well as a new treatment combination for patients suffering from high blood pressure and other cardiovascular diseases. Although scientific evidence on the role of microRNAs in RAAS signaling is scarce, the few published studies on circulating microRNAs in patients with coronary artery diseases do indeed indicate that some of these circulating microRNAs may be used as biomarkers of therapeutic approaches targeting RAAS and cardiovascular diseases.

## Figures and Tables

**Table 1:** MicroRNAs affected by RAAS effectors.

Effector	MicroRNA target gene	Reference
Angiotensin II		
miR-155	ATR1, eNOS, *α*-SMA, NF-*κ*B, AP-1	[11–14, 16, 48]
↓ miR-29-b	TGFbeta, Col 1A, *α*-SMA	[18, 24, 33–35]
↓ miR-483-3p	AGT, ACE-1, ACE-2, AT2R	[29]
↓ miR-129-3p	FAK, MMP-2, MMP-9	[23]
**↑ **miR-132/212	AT1R, MSK, G*αβ*/ERK1/2	[19, 21]
↓ miR-34	ANP, *β*-MHC	[46, 49]
miR-766	Cyp11B2	[25]
miR-16	Ang II, CCDN1, CCDN2, CCDNE	[47]

Note: ↓: decreased expression level; ↑: increased expression level.

**Table 2:** MicroRNAs affected by RAAS inhibitors.

Inhibitor	MicroRNA target gene	Reference
Aliskiren		

↓ miR-106-5p	EGFR/AKT/mTOR, ACE	[27]
↓ miR-27a-3p	EGFR/AKT/mTOR, ACE	[26–28, 38]
↓ miR-18b-5p	EGFR, ACE	[29]
↓ miR-155	AT1R	

Candesartan		

↑ miR-483-3p	AGT, ACE-1, ACE-2, AT2R	[26, 38]
↓ miR-132/122	Ang II	[38]
↓ miR-29b	Col1A, Col3A1	[26, 38]
↓ miR-212	AT2R	

Telmisartan		

↑ miR-31		[30]
↑ miR-181a	TNFalpha	[30]
↑ miR-16	VEGF	[30]
↑ miR-143/145	KLF4, KLF6, ACE-2	[30]
↓ miR-146a/b	TRAF6, KLF4, TLR4	[31]

Atorvastatin		

↓ miR-146a/b	TRAF6, KLF4, TLR4	[31]
↓ miR-221/222	p27, p57	[50]

Enalapril		

↑ miR-31		[30]
↑ miR-181a	TNFalpha	[30]
↑ miR-145	KLF4, KLF6, ACE-2	[30]
↑ miR-16	VEGF, CCND1, CCND2, CCNE	[30, 47]

Captopril		

↑ miR-16	VEGF	[30, 47]
↑ miR-19b	*β*MHC	[47, 51]
↑ miR-20b		[47]
↑ miR-93		[47]
↑ miR-106b		[47]
↑ miR-223		[47]
↑ miR-423-5p		[47]

Note: ↓: decreased expression level; ↑: increased expression level.

## Abbreviations

AS: Aldosterone synthase
ASI: Aldosterone synthase inhibitors
ANG: Angiotensin
ACE: Angiotensin converting enzyme
ARB: Angiotensin receptor blockers
CRP: C-reactive protein
CVD: Cardiovascular disease
DRI: Direct rennin inhibitors
EPC: Endothelial progenitor cells
ERK: Extracellular receptor kinase
EGFR: Epidermal growth factor receptor
eNOS: Endothelial nitric oxide synthase
EDHF: Endothelium-derived hyperpolarizing factor
ET-1: Endothelin 1
IL-1*β*: Interleukin 1 beta
IL-6: Interleukin 6
ICAM-1: Intracellular cell adhesion molecule 1
IGF: Insulin growth factor
MCP-1: Monocytes chemoattractant protein 1
MIP-1: Monocytes inflammatory protein 1
miR: MicroRNA
MRA: Mineralocorticoid receptor antagonist
NF-*κ*B: Nuclear factor kappa B
NO: Nitric oxide
PPAR*γ*: Peroxisome proliferators-activated receptor gamma
RAAS: Renin-angiotensin-aldosterone system
TGF*β*: Transforming growth factor beta
TNF*α*: Tumor necrosis factor alpha
VCAM-1: Vascular cell adhesion molecule 1.
